# Development of Mathematical Models for the Analysis of Hepatitis Delta Virus Viral Dynamics

**DOI:** 10.1371/journal.pone.0012512

**Published:** 2010-09-16

**Authors:** Bruno C. de Sousa, Celso Cunha

**Affiliations:** 1 Centre for Malaria and Tropical Diseases, Associated Laboratory, Unit of Epidemiology and Biostatistics, Instituto de Higiene e Medicina Tropical - Universidade Nova de Lisboa, Lisbon, Portugal; 2 Centre for Malaria and Tropical Diseases, Associated Laboratory, Molecular Biology Unit, Instituto de Higiene e Medicina Tropical - Universidade Nova de Lisboa, Lisbon, Portugal; Albert Einstein College of Medicine, United States of America

## Abstract

**Background:**

Mathematical models have shown to be extremely helpful in understanding the dynamics of different virus diseases, including hepatitis B. Hepatitis D virus (HDV) is a satellite virus of the hepatitis B virus (HBV). In the liver, production of new HDV virions depends on the presence of HBV. There are two ways in which HDV can occur in an individual: co-infection and super-infection. Co-infection occurs when an individual is simultaneously infected by HBV and HDV, while super-infection occurs in persons with an existing chronic HBV infection.

**Methodology/Principal Findings:**

In this work a mathematical model based on differential equations is proposed for the viral dynamics of the hepatitis D virus (HDV) across different scenarios. This model takes into consideration the knowledge of the biology of the virus and its interaction with the host. In this work we will present the results of a simulation study where two scenarios were considered, co-infection and super-infection, together with different antiviral therapies. Although, in general the predicted course of HDV infection is similar to that observed for HBV, we observe a faster increase in the number of HBV infected cells and viral load. In most tested scenarios, the number of HDV infected cells and viral load values remain below corresponding predicted values for HBV.

**Conclusions/Significance:**

The simulation study shows that, under the most commonly used and generally accepted therapy approaches for HDV infection, such as lamivudine (LMV) or ribavirine, peggylated alpha-interferon (IFN) or a combination of both, LMV monotherapy and combination therapy of LMV and IFN were predicted to more effectively reduce the HBV and HDV viral loads in the case of super-infection scenarios when compared with the co-infection. In contrast, IFN monotherapy was found to reduce the HDV viral load more efficiently in the case of super-infection while the effect on the HBV viral load was more pronounced during co-infection. The results suggest that there is a need for development of high efficacy therapeutic approaches towards the specific inhibition of HDV replication. These approaches may additionally be directed to the reduction of the half-life of infected cells and life-span of newly produced circulating virions.

## Introduction

Hepatitis delta virus (HDV) is considered to be a satellite virus of the hepatitis B virus (HBV). HDV co-infects or super-infects liver cells already infected with HBV resulting in an higher risk of cirrhosis and fulminant hepatitis, as well as increased liver tissue damage [1], [2]. Hepatitis delta virus contains a ribonucleprotein core which includes a 1.7 Kb circular single-stranded RNA genome and several copies of the only virus encoded protein, the so called delta antigen (reviewed in Taylor, [3]). The clinical association between HDV and HBV is due to the fact that the outer envelope of HDV consists of the surface antigens coded by the HBV genome (HBsAgs) which are necessary for virion maturation and release from the cells (reviewed by Taylor in [4]). Therefore, productive HDV infection occurs only in the presence of HBV.

It is widely accepted that the clinical course of super-infection and co-infection displays distinct features. In most cases, super-infection of chronic HBV patients results in the development of chronic HDV infection. In general, the clinical course of HDV super-infection starts with an acute phase which is followed by the development of chronicity, and finally the elimination of HDV and HBV. During the acute phase of infection, an active replication of HDV is observed whilst HBV replication is partially suppressed. The following chronic phase is characterized by a decrease in HDV replication which is accompanied by a subsequent increase in HBV replication [5]. It is estimated that about 70% of super-infected patients will progress from acute to chronic disease. Additionally, 60–79% of chronic HDV patients will further develop cirrhosis. This rate is 3 times higher than that found in HBV or HCV infected patients alone [6]. According to Fattovich et al. [7], HDV super-infection leads to a 3 times greater increase in risk of hepatocellular carcinoma and twice greater rates of mortality in patients with compensated cirrhosis. In HDV and HBV co-infections, the clinical course is similar to that observed during acute HBV infection [8], [9].

There is no specific treatment for HDV infection. The most common therapeutic approach is based on the administration of interferon-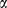. However, the clinical response is variable, and in most cases reversible upon interruption of treatment [10]–[12]. The concomitant use of antiviral drugs like ribavirin or lamivudine, showed no significant benefits in the treatment of hepatitis delta patients [13]–[15]. Although these drugs may have some inhibitory effect on HBV replication, they do not suppress HDV replication probably due to the fact that HBsAgs expression, at least in part, seems not to be affected.

Vaccination against HBV protects individuals against HDV co-infection. Although vaccination programs led to a considerable reduction in both HBV and HDV prevalence, the two viruses are still endemic in a number of regions, namely the Amazon basin, and some African and Asian countries [16]. It is estimated that worldwide about 350 million people are infected with HBV of which 5–10% are also co-infected with HDV. HDV and HBV share the same routes of infection affecting individuals of all age groups. The most frequent routes of transmission are the sexual contact and the direct contact with blood or blood products from infected carriers [17], [18].

The use of mathematical models to study dynamics of virus infections may represent a powerful approach to simulate the course of infection and predict the potential response to different therapies. They have been previously developed for a number of pathologies including HIV, HBV, and HCV [19]–[21]. More recently, the mathematical simulation of the spread of HDV and HBV in a population was reported [22]. However, a mathematical model to study HDV and HBV dynamics in infected individuals is still lacking. In this initial work we report the development of mathematical models to simulate the dynamics of HDV and HBV, both during co-infection and super-infection, in the presence and absence of any therapy. Data are presented concerning the number of predicted infected cells and viral load along the period of infection.

## Methods

To model for the viral dynamics of HDV we considered the following six variables:

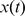
 the number of uninfected cells at time 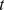,

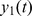
 the number of HBV infected cells at time 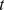,

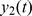
 the number of HDV infected cells at time 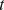,

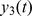
 the number of infected cells with both HBV and HDV at time 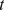,

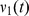
 the HBV viral load at time 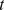,

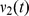
 the HDV viral load at time 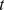.

**Figure 1:**
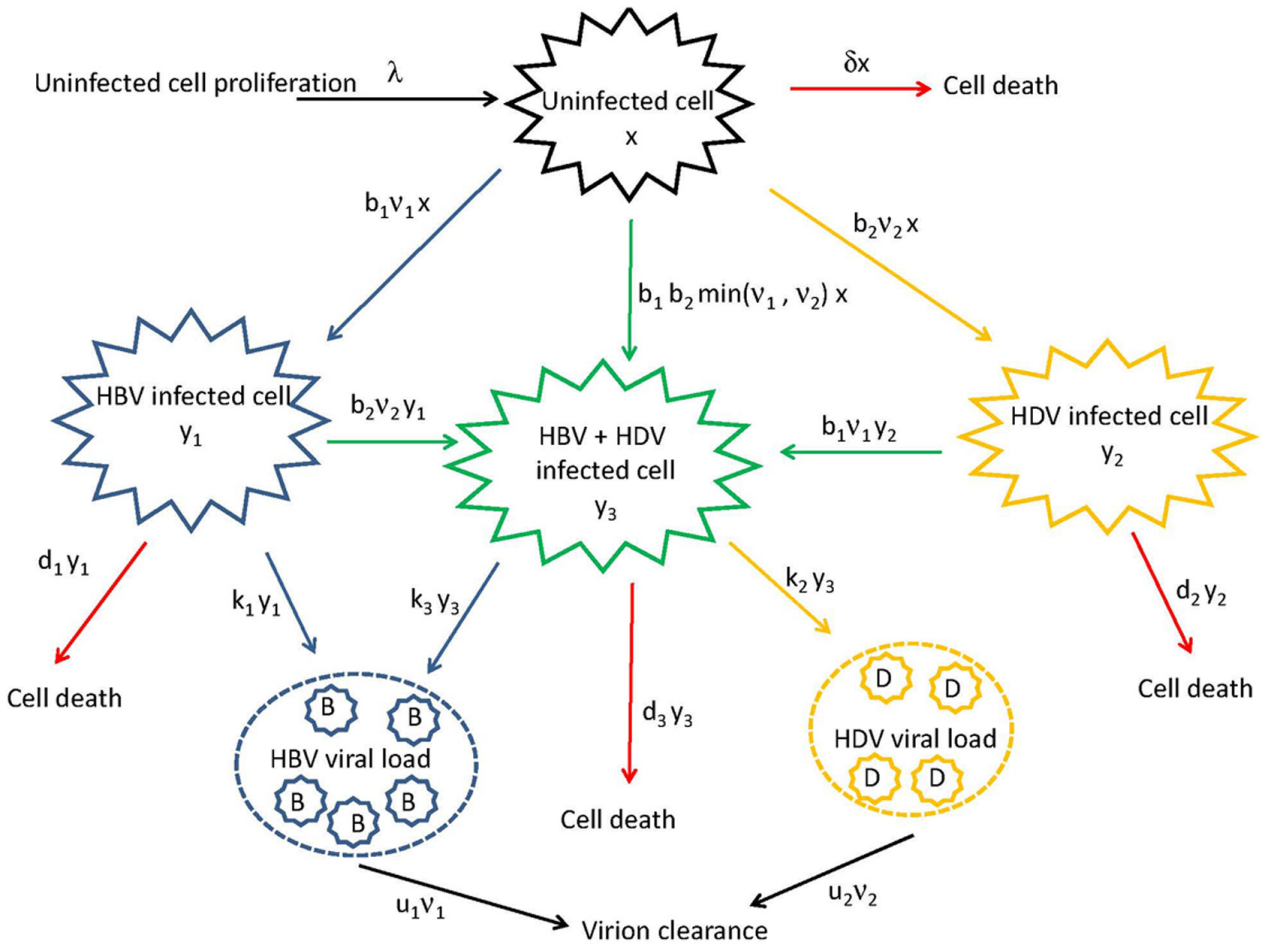
Diagram representing the HDV viral dynamics within an individual.

The model representing the hepatitis delta virus (HDV) viral dynamics can be represented in the diagram in Figure 1, followed by a description of the variables and parameters represented in this diagram.

he change in the number of uninfected cells 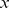 at a certain moment in time will depend on the constant rate at which these cells are generated, 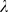; the number of deaths at that time, which is proportional to the constant death rate of uninfected cells, 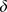; and the number of infected cells lost by infection with HBV and HDV which are proportional to the constant infection rates of HBV and HDV, 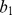 and 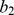, respectively, and to the HBV and HDV viral loads at that time 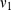 and 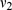, respectively.

With respect to the change in the number of 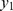, HBV infected cells, at a certain moment in time, its dependency involves the constant infection rate of HBV, 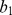, the number of uninfected cells 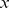 and the HBV viral load 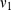 at that time. Also, the constant death rate of HBV infected cells, 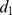; and the infected cells with HBV that were co-infected with HDV. The later, proportional to the constant infection rate of HDV, 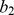, the number of HBV infected cells, 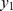, and the HDV viral load, 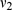, at that time.

Similarly, the change in the number of HDV infected cells at a certain time, 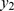, will depend on the constant infection rate of HDV, 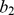, the number of uninfected cells 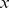 and the HDV viral load 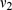 at that time; the constant death rate of HDV infected cells, 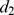; and the infected cells with HDV that were co-infected with HBV at that time which is proportional to the constant infection rate of HBV, 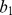, the number of HDV infected cells, 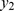, and the HBV viral load, 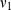, at that time.

The change in the number 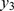 of infected cells with both HBV and HDV at a certain time will depend on the number HBV infected cells that are infected with HDV at that time; the number HDV infected cells that are infected with HBV at that time; and the number of uninfected cells that are infected simultaneously with HBV and HDV, 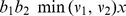, proportional to the constant infection rates of HBV and HDV and the viral loads of HBV and HDV.

The change of HBV viral load at a certain time will depend on the constant HBV virion clearance rate 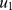, and from both sources of viral production, i.e. cells that are infected only with HBV and with both HBV and HDV. From cells only infected with HBV, the viral load is proportional to the constant HBV viral production rate 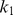 and the number of HBV infected cells at that time 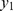. From cells infected with both viruses, the viral load is proportional to the constant HBV viral production rate 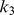 and the number of HBV and HDV infected cells at that time 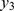.

As for the change in HDV viral load, the dependency of this viral load will also come from the constant HDV virion clearance rate 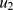, but now the viral production will only come from cells that are infected with both HBV and HDV. Therefore, the change of HDV viral load at a certain moment in time is proportional to the constant HDV viral production rate 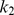 and the number of HBV and HDV infected cells at that time 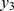.

As a result of the above, the model for the viral dynamics in an individual infected with HBV and HDV can be expressed as the following system of six differential equations modeling the changes in 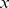, 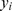 (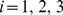) and 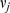 (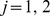):
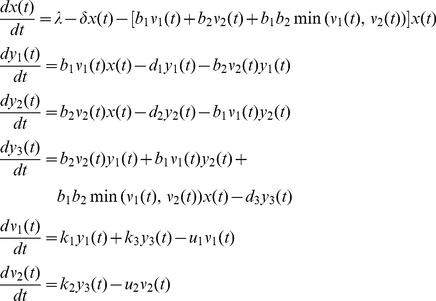
(1)


### Solving the Mathematical Model

The system of differential equations in (1) needs to be solved numerically. We used the software *Matlab 7.5* [23] where we simulated the behavior of our proposed model considering two different kinds of infection: co-infection and super-infection. Co-infection occurs when an individual is simultaneously infected by HBV and HDV, while super-infection occurs in persons with an existing chronic HBV infection. Therefore, for a co-infection scenario the viral dynamics can be modeled simply by the equations in (1), while for the super-infection scenario, we assumed that the individual is first infected with HBV and that the infection of HDV occurs after 200 days (d) of being infected with HBV. Thus, before 200 d, the model (1) is simplified by the following model (2) since only the infection with HBV has occurred.
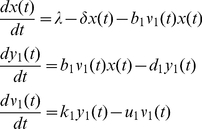
(2)


In both scenarios, and to obtain a numerical solution for the viral dynamics expressed by the models (1) and (2), we will need to provide some initial values for the functions 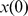, 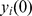 (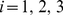) and 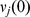 (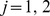) and the 12 constant rates involved in these models. Unless mentioned otherwise, we will now present the assumptions considered in all simulations throughout this study. The values considered are summarized at the end of this section in Table 1.

**Table 1:** Parameters considered in this study and corresponding values.

Parameter	Values	Reference
Liver cell number	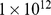	[24]
Blood volume	6000 mL	[24]
Cell division rate	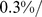 day	[24], [26]
Liver cells half-life	231 days	[24], [26]
Virion life-span in plasma	15–92 hours	[37], [28]
Infected cells half-life	10–100 days	[37], [28]
Infection rate	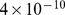 mL/copies per day	[24]
Viral production rate	6.24/day	[24]
Virion clearance rate	6.24/day	[24]
Mean viral load in equilibrium	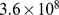 copies/mL	[24]
Infected cells number	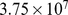 cells/mL	[24], [29]
Clearance constant rate	0.65/day	[24]

All the values for 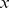, 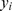 (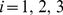) and 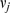 (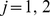) are expressed in number of copies per milliliter of blood. We will consider that at time 0 there are no cells infected with HBV or HDV, i.e. 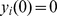 for 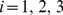. Let us assume three different levels of viral load at the time of infection, 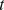: a low level of infection with 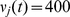 copies/mL; a medium level of infection with 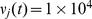 copies/mL; and a high level of infection with 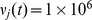 copies/mL for 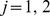. For co-infection, we have 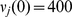 for 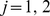, and for the super-infection we have 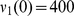, 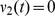 for 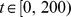, and 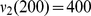. Finally, the number of uninfected cells in an adult has been estimated to be equal to 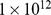, and considering that the average blood volume of an adult is equal to 6000 mL [24], the number of uninfected cells per mL can be estimated by 
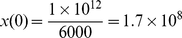
 cells/mL [24].

The constants in our study were based on the work by Tsiang and Gibbs in [24] and summarized in what follows. From [25] and [26] approximately 0.3% of the liver cells in rats go through mitosis every day. Assuming the same for humans, the constant rate at which uninfected cells are generated, 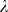, can be estimated by 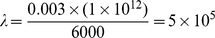 cells/mL per day. Since before infection the liver is in equilibrium, i.e. the number of uninfected cells is assumed to be constant. Therefore, we have that 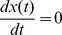, meaning that, at 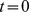, we have 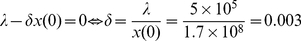 d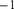. In terms of half-lives 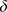 is equal to 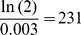 days.

Regarding the virion clearance rates 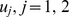 and the cell death rates 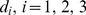, it is easier to interpret them as their inverse 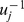 and 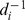, representing the mean life-spans of HBV and HDV virion in plasma (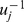) and the mean life-spans of productively infected cells with HBV, HDV and both HBV and HDV (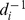), respectively. Since there is very little known about the dynamics of the hepatitis delta virus, we considered not only what it is known in multiple studies with HBV and HDV patients [5], [24], [27], but also the opinions of clinicians and biologists that suggested possible values of 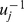 in the range of 15 to 92 hours, and for 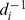 values between 10 days and 100 days [28]. In what follows, we consider the following set of values 
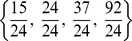
 and 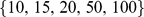 days for 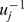 and 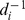, respectively.

In terms of the 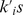, the viral production rates of HBV from HBV infected cells (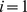), of HDV (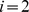) and HBV (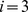) from cells infected with both HBV and HDV, it is believed that, at least during some periods of infection, HDV replication will have an inhibitor effect on the production of mature HBV particles; therefore, for our study we considered that 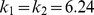 d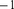, and when the infection of HDV occurs, the viral production rate of HBV from cells infected with HBV and HDV, 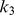, is equal to 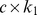 d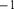 with 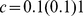 (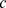 varying from 0.1 to 1 with increments of 0.1), where 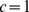 represents no inhibition effect. The value of 6.24 d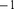 for 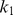 was suggested by Tsiang and Gibbs in [24] considering the fact that, when infection reaches an equilibrium, we have that 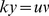, i.e. the viral production rate from an infected cell is approximately equal to the virion clearance. The mean viral load in the equilibrium was considered to be 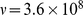 copies/mL, the clearance constant rate 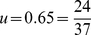 and the number of infected cells to be approximately 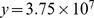 cells/mL. This value was obtained considering the work of Bianchi, *et al.* [29] where it was reported that approximately 5–40% of hepatocytes are infected in chronic HBV patients. Tsiang and Gibbs in [24] considered then the mean value of 22.5% and calculated 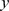 as 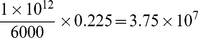.

The infection rates, 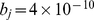 mL/copies per day, for 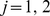, were also suggested by Tsiang and Gibbs in [24]. They were determined such that the peak of the primary viremia occurred 56 days after the infection of an individual.

We also assumed that when the super-infection occurs there is an extra HBV inoculum at that moment. In our model we considered values for extra HBV inoculum of the order 400, 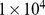 and 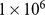 copies/mL, equals to the initial viral load of HBV.

In the next section we will discuss in detail the results obtained from the simulations in *Matlab 7.5*. Although we present only the graphs considering initial viral loads of 400 copies/mL, the viral dynamics for all other scenarios is described in this study as well.

## Results

### The super-infection results

The values of HDV life-span in plasma of patients and of life-span of HDV infected liver cells are, to our knowledge, unknown. Although assuming that these values should not be substantially different from those reported for HBV [5], [24] we first decided to perform a sensitivity analysis of the influence of these parameters on the dynamics of infection.

**Figure 2:**
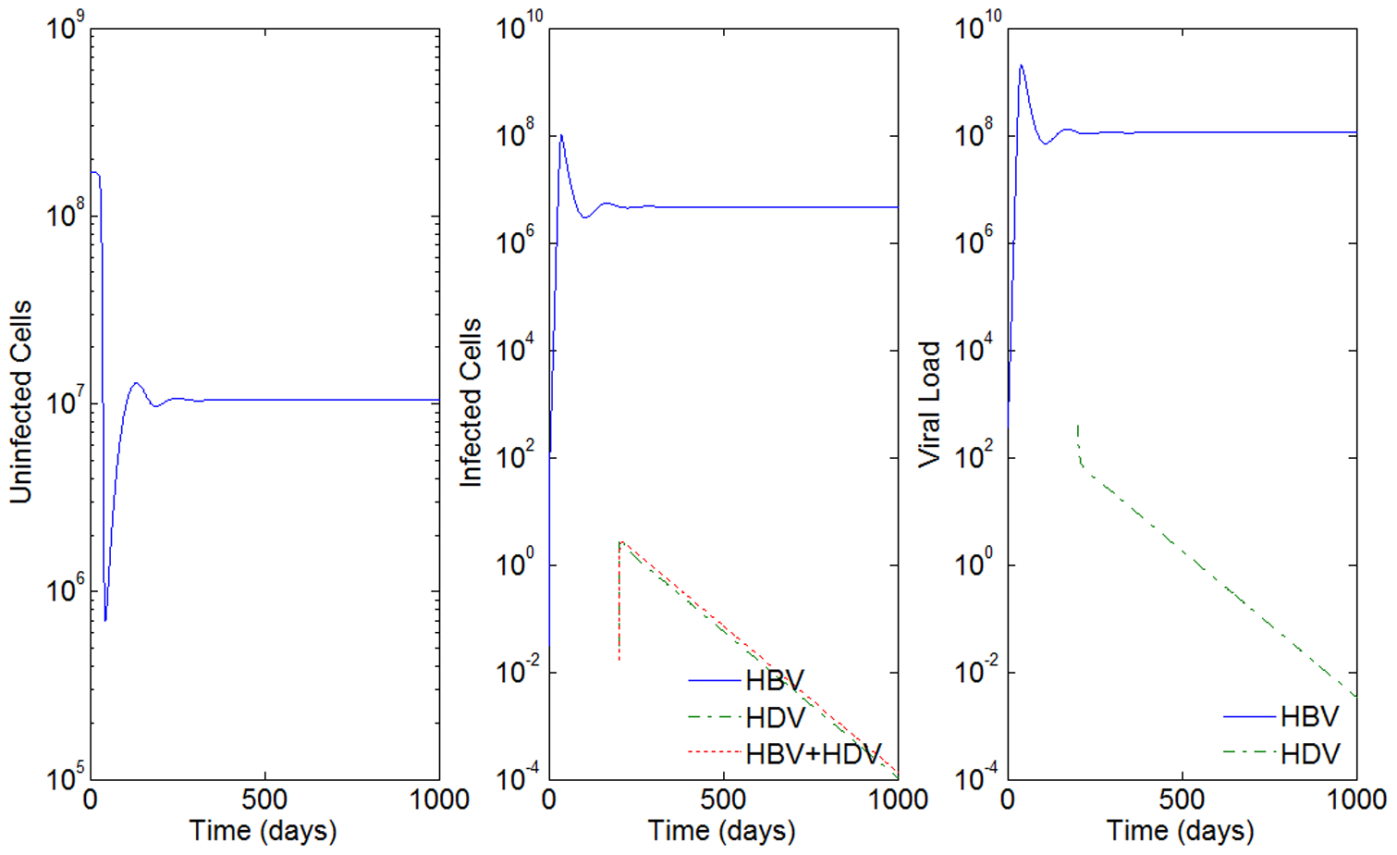
HBV and HDV viral dynamics. HBV Number of uninfected cells (left), infected cells (middle), and viral loads (right). Initial viral loads of 400 copies/mL, 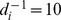 days, 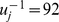 hours, and a 10% inhibition of the HBV viral production in cells infected with HBV and HDV.

For 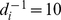 days, none of the values considered for 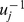
's produced scenarios for the super-infection that are clinically observed since they would predict a spontaneous HDV clearance soon after infection. Consider, for example, Figure 2 where 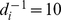 days and 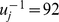 hours. Unfortunately, the viral load of HDV and the number of infected cells do not disappear naturally, as suggested in this figure.

**Figure 3:**
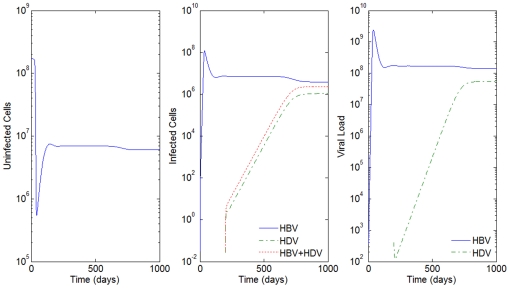
HBV and HDV viral dynamics. Number of uninfected cells (left), infected cells (middle), and viral loads (right). Initial viral loads of 400 copies/mL, 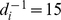 days, 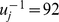 hours, and a 10% inhibition of the HBV viral production in cells infected with HBV and HDV.

**Figure 4:**
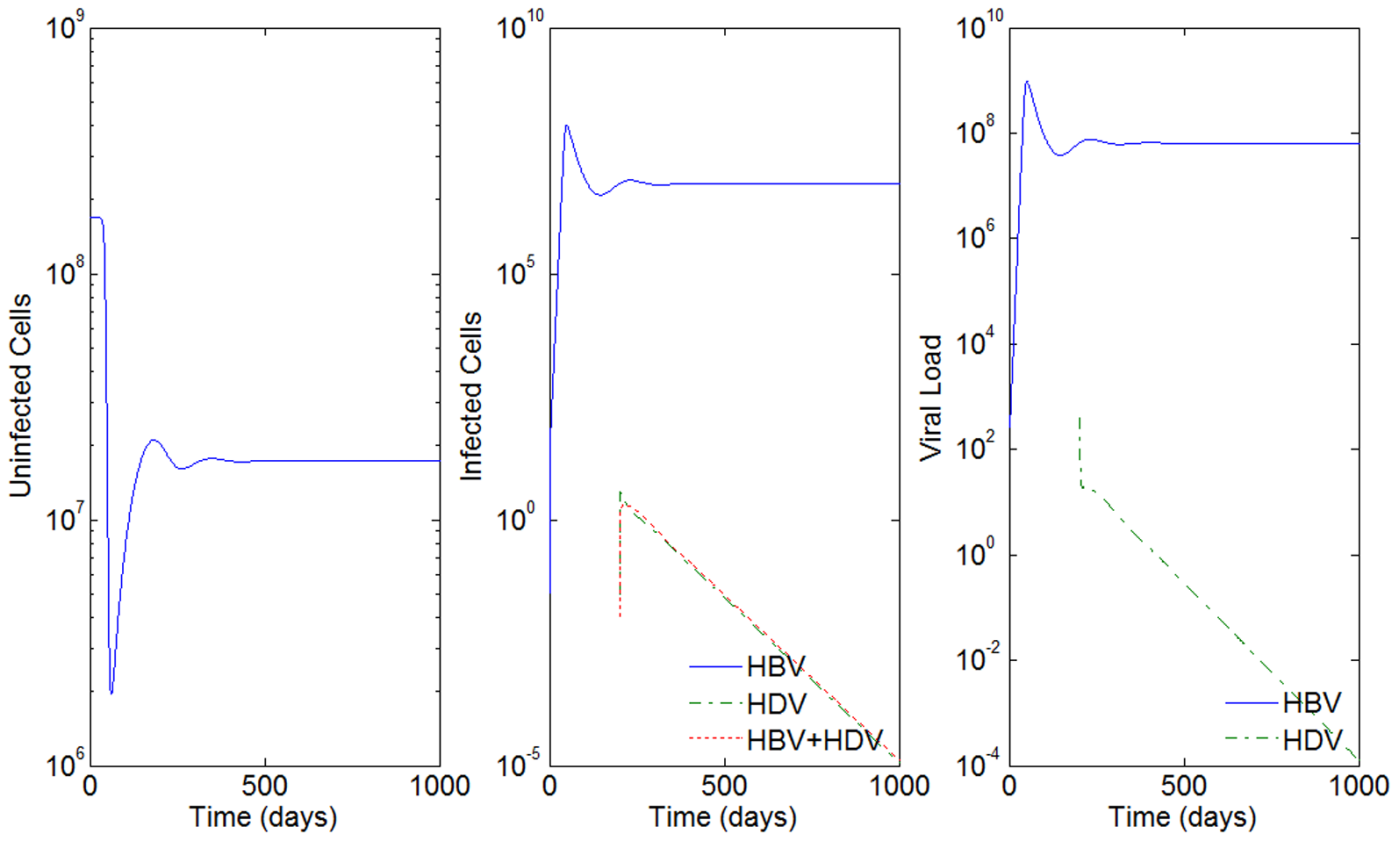
HBV and HDV viral dynamics. Number of uninfected cells (left), infected cells (middle), and viral loads (right). Initial viral loads of 400 copies/mL, 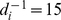 days and 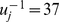 hours, and a 10% inhibition of the HBV viral production in cells infected with HBV and HDV.

By increasing the mean life-spans of infected cells by 5 days, i.e 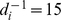 days, and maintaining 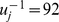 hours, we can observe the dynamics of the super-infection in Figure 3. Nevertheless, a biologically irrelevant scenario occurs again for all other values of 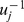. See the result of the simulations for 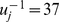 hours in Figure 4.

For values of 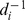
's of 50 and 100 days, all the results were compatible with reported clinical pictures. What the simulations suggest is that the smaller the values of mean life-spans of productively infected cells, 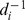, the higher the values of mean life-spans of HBV and HDV virion in plasma, 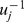, needed in order to obtain biologically relevant scenarios. Similar results were obtained when the effect of inhibition of HBV replication by HDV was tested with different values of 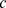 up to 1. We decided to create a finer grid for the values of 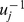
's to estimate the approximate minimum value for the mean life-spans of HBV and HDV virion in plasma in order to obtain possible biological observed scenarios. The results can be seen in Table 2.

**Table 2:** Estimated minimum values for 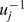.

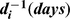	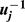 (hours)
10	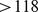
15	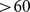
20	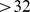

**Figure 5:**
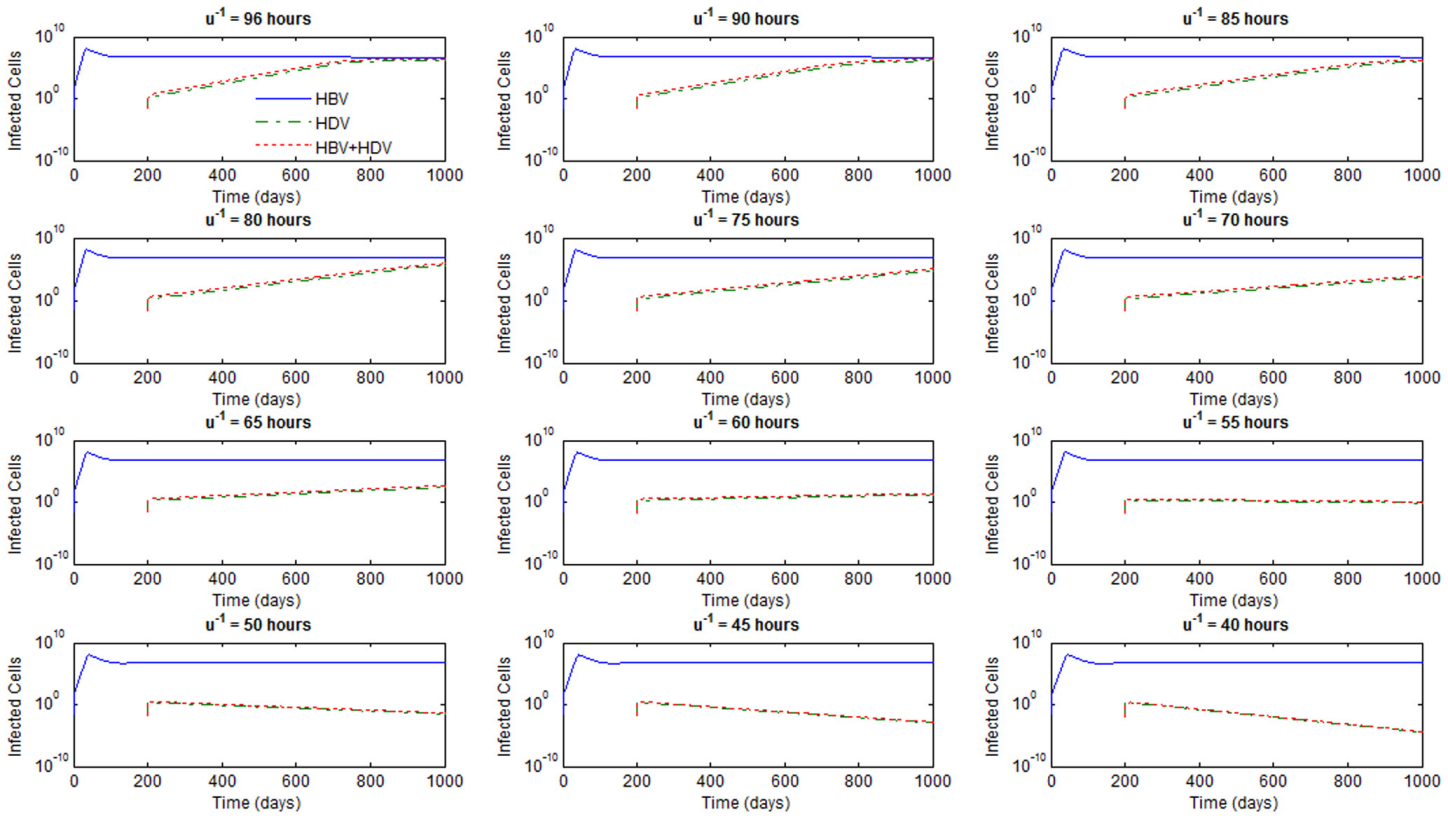
HBV and HDV viral dynamics. Number of infected cells with HBV, HDV and with both HBV and HDV for different values of 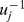
's in hours and 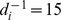 days.

The results obtained for estimating the minimum values for 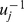
's in order to obtain observable biological scenarios were quite similar, independent of the initial viral loads for HBV and HDV and the different values of the inhibition factor 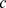 for the viral production rate of HBV from a cell infected with HBV and HDV. In Figure 5 we see an example of the grid values considered for 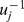
's, with 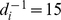 days, initial viral loads of 400 copies/mL, and for 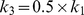. It is very clear that values of 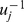
's smaller than 60 hours generate a scenario that is not observed in patients.

**Figure 6:**
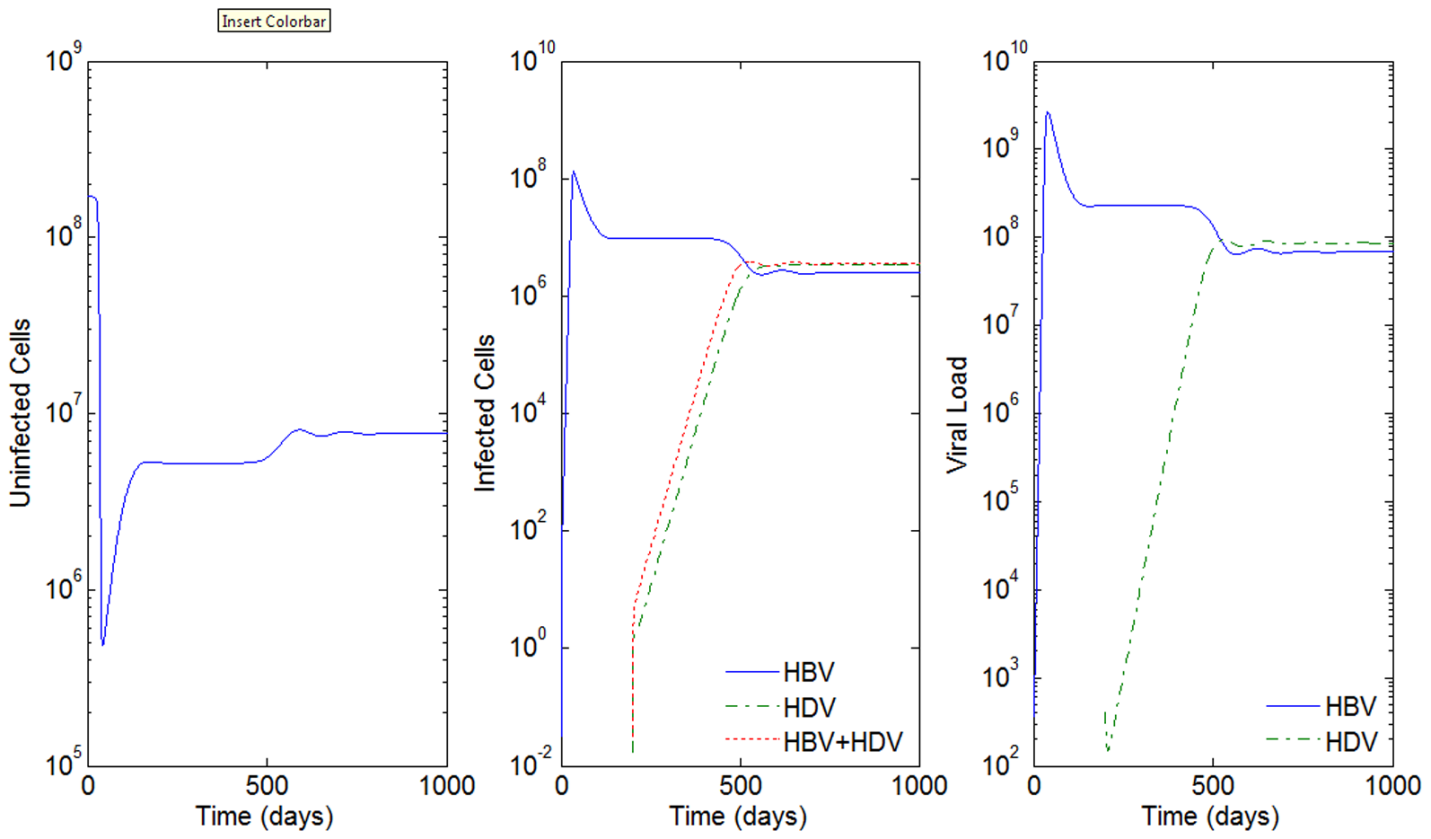
HBV and HDV viral dynamics. Number of uninfected cells (left), infected cells (middle), and viral loads (right). Initial viral load of 400 copies/mL, 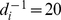 days and 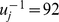 hours, and a 90% inhibition of the HBV viral production in cells infected with HBV and HDV.

**Figure 7:**
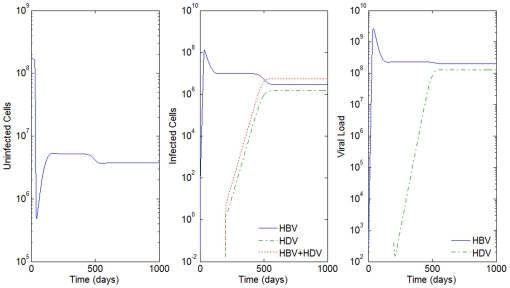
HBV and HDV viral dynamics. Number of uninfected cells (left), infected cells (middle), and viral loads (right). Initial viral loads of 400 copies/mL, 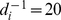 days and 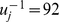 hours, and no inhibition of the HBV viral production in cells infected with HBV and HDV.

The particular effect of the inhibition factor 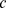 of the HBV viral production rate from an infected cell with HBV and HDV, 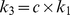, can only be observed for large values of the mean life-spans of productively infected cells. For example, for 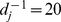 days and 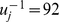 hours, the higher the value of 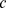 (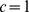 representing no inhibition), the closer the viral loads of HBV and HDV are. A more unstable behavior of the viral loads is observed for greater values of the inhibition (small values of 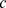). The least number of infected cells is always observed for those only infected with HBV, followed by the ones only infected with HDV, and lastly the HBV and HDV infected cells. For higher values of inhibition, say, greater than 80% (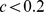), the number of cells infected only by HDV surpasses the number of cells infected with both HDV and HBV. A similar picture is observed for the corresponding values of free viral load. In Figure 6 and Figure 7 we can observe this behavior where the inhibition varies from 90% (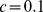) to no inhibition, respectively.

**Figure 8:**
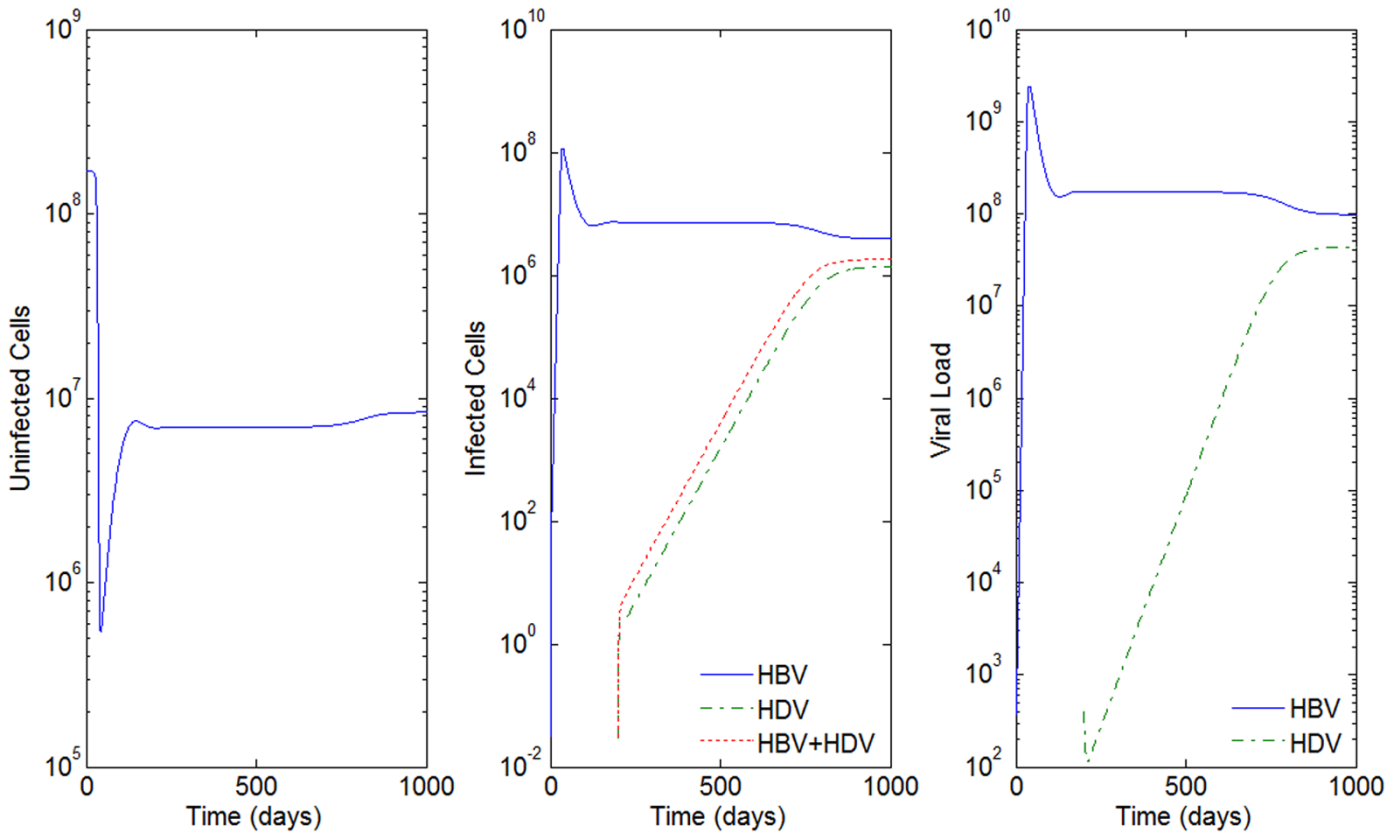
HBV and HDV viral dynamics. Number of uninfected cells (left), infected cells (middle), and viral loads (right). Initial viral loads of 400 copies/mL, 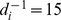 days, 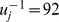 hours and 90% inhibition of the HBV viral production in cells infected with HBV and HDV.

Notice that for 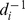
's equal to 15 days and 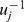
's equal to 92 hours, there is no effect of the parameter 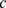 on the results of the model. Notice how close Figure 8 is to Figure 3, where we had 90% and 10% of inhibition of the HBV viral production in cells infected with HBV and HDV, respectively. In this case, we see from Figure 8 that the HDV viral load is always smaller than the HBV viral load, and that the number of infected cells is highest for HBV only infected cells, followed by the ones infected with both HBV and HDV, and lastly by the number of cells only infected with HDV.

Since all HDV infected patients are also infected with HBV, we also considered the scenario of extra HBV inoculum occurring concomitantly with the HDV infection. In our simulations we considered this extra HBV inoculum to be equal to the viral load of HDV. The results obtained were the same whether this supplement of HBV occurs or not.

For all biological relevant scenarios, the simulations here reported predict the existence of a peak of viral load and number of infected cells in the beginning of infection. This is particularly noticeable in the case of HBV. A similar observation was reported by Tsiang and Gibbs [24] when modeling HBV infection alone. This feature may represent an artifact of the model although in woodchuck hepatitis virus (WHV), peaks of viremia in infected experimental animals were observed during the first weeks of infection (see for instance [30]).

### The co-infection results

Co-infection occurs when the individual is infected with HBV and HDV at the same time. The results obtained for the co-infection using model (1) are, in general, very similar to the ones obtained for super-infection. The differences are essentially based on the speed at which the individual reaches the peak of infection and certain behaviors when the infection is in equilibrium. Next, we will show some of the results obtained for co-infection where we try to illustrate the similarities and the differences between these two scenarios.

**Figure 9:**
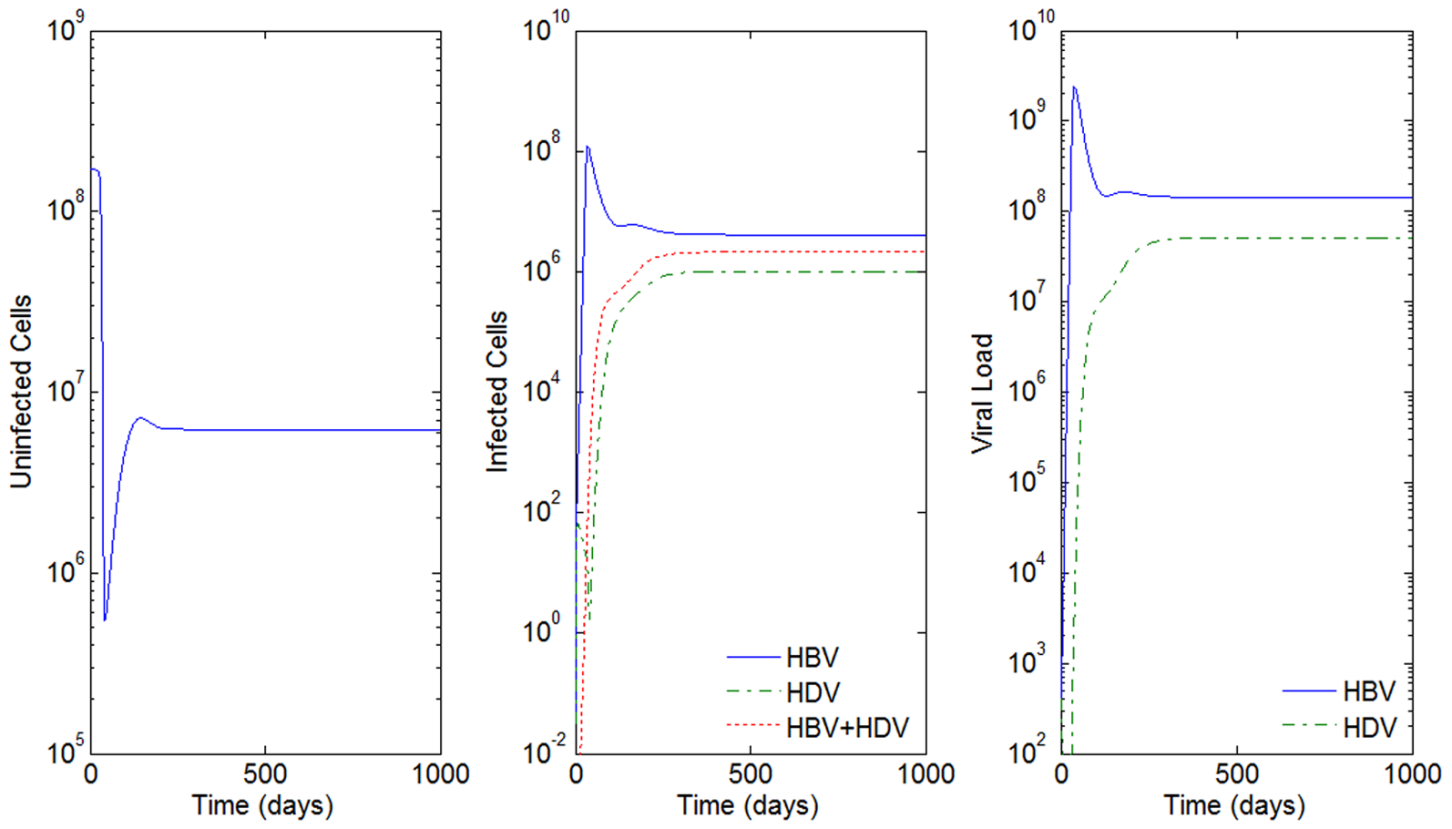
HBV and HDV viral dynamics. Number of uninfected cells (left), infected cells (middle), and viral loads (right). Initial viral loads of 400 copies/mL, 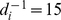 days, 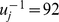 hours, and a 10% inhibition of the HBV viral production in cells infected with HBV and HDV.

Consider the example in Figure 3 where 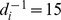 days, 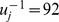 hours, initial viral loads of 400 copies/mL and a 10% inhibition of the HBV viral production in cells infected with HBV and HDV. In Figure 9 we simulate the co-infection behavior with 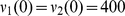 copies/mL, i.e. the infection of HBV and HDV occurs at the same time, 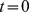.

By comparison with Figure 3, we see that while in super-infection it takes approximately 550 days from the day of infection with HDV to reach an equilibrium, in the co-infection case, this equilibrium is reached after 200 days. In all the examples analyzed, the slope of the line representing the viral load growth and the number of infected cells with HDV and with both HBV and HDV is higher for the co-infection.

**Figure 10:**
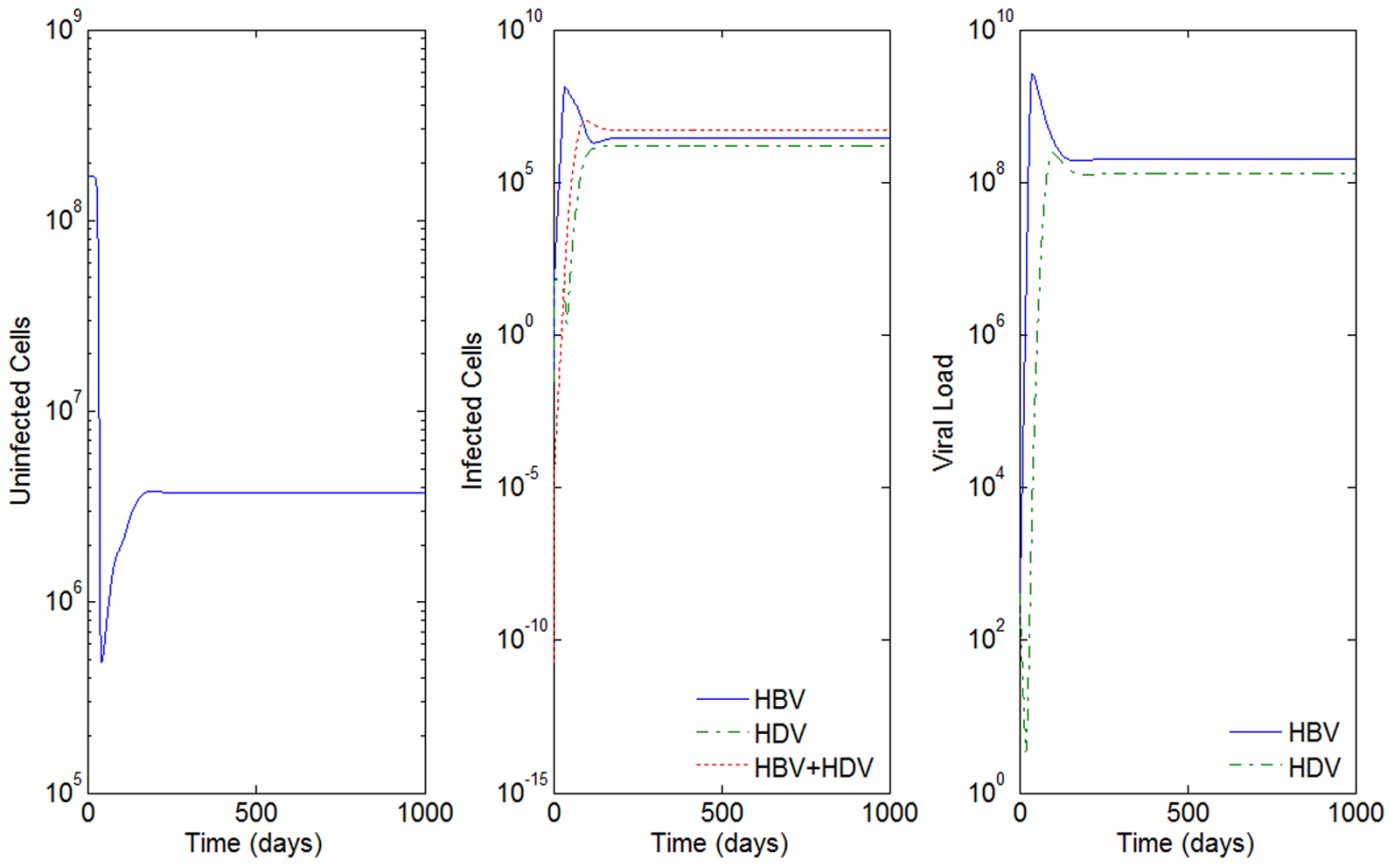
HBV and HDV viral dynamics. Number of uninfected cells (left), infected cells (middle), and viral loads (right). Initial viral loads of 400 copies/mL, 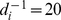 days, 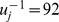 hours, and no inhibition of the HBV viral production in cells infected with HBV and HDV.

It is also believed that for HBV and HDV co-infection the clinical course does not differ from that observed in patients infected HBV alone [5]. In situations of no inhibition the major difference for co-infection is the stability of the viral load when the equilibrium is reached. Compare both the right graphs in Figure 7 and Figure 10 for 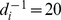 days, 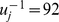 hours. In co-infection we see that the viral loads for HBV and HDV have a very similar and rapid behavior, and that the higher number of infected cells are represented by the cells infected with both viruses, followed by the ones infected with HBV, and lastly the ones infected only with HDV, as can be observed in Figure 10. This contrasts with the very slow growth during the evolution of the disease when super-infection occurs, resulting in a different behavior of the number of infected cells. Recall that for the super-infection case, the smaller number of infected cells occurred for cells infected only with HBV.

From the results obtained in this study, we can say that the development of the disease in super-infection is slower than in co-infection, suggesting that for super-infection a larger window of time is available before the beginning of therapy. Might that imply different reactions when therapies are applied? This will be explored in the following section.

### The Model considering Antiviral Therapy

Antiviral therapy will have an impact on HDV viral dynamics depending on whether it is aimed at eliminating the virus itself including through modulation of the immune system (i.e. interferon-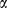), or inhibiting HBV free virion production (i.e. lamivudine or ribavirine). Although there is no specific treatment for HDV infection, there has been some recent successful stories in treating patients [31]. As mentioned before, the most common therapeutic approach is based on the administration of peggylated interferon-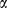 which helps promoting virus clearance. However, the clinical response is variable, and in most cases reversible upon interruption of treatment [10]–[12]. The concomitant use of antiviral drugs like ribavirin or lamivudine, which is believed to reduce the production rate of HBV free virions that are released from infected cells in the blood, showed no significant benefits in the treatment of hepatitis delta patients [13]–[15]. Although these drugs may have some inhibitory effect on HBV replication, they do not suppress HDV replication, probably due to the fact that HBsAgs expression is not significantly affected.

Let 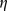 be the efficacy of inhibiting new virus infections as a consequence of virus clearance, and 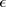 the efficacy of inhibiting viral production from infected cells, with both 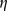 and 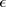 in the interval 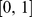. The antiviral impact from the different types of therapies can then be introduced in the initial model ((1)) as follows (changes in **bold**):
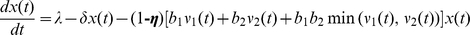


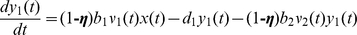


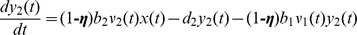


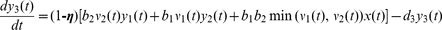


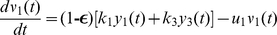


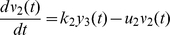



Although the mechanism of the action of interferon-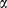, IFN, in HDV patients is not clearly understood [32], some studies report improvements in patients, with IFN efficacy as high as 90% (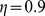) [33]. Although lamivudine (LMV) does not have a direct effect on HDV viral production 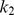 (notice the absence of 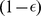 in the equations above) its effect on the viral production of HBV will also have an effect on the HDV viral dynamics. Studies such as [28] show efficacy levels of therapies based on LMV varying between 90% to 99% (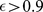) for the HBV infection.

With this in mind, we considered 5 different scenarios of antiviral therapy responses for super-infected and co-infected individuals in our simulations: two with monotherapies with LMV and IFN alone and three others with LMV and three different efficacy levels of IFN, i.e. 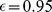 and 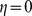 (LMV antiviral therapy), 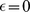 and 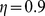 (IFN antiviral therapy) and 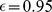 with 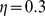, 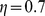 and 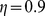 (LMV and IFN antiviral therapy). These last three scenarios represent ones where LMV is very efficient, but with different patient's response levels to IFN. Although the antiviral therapy responses for co-infected individuals were less pronounced, the results were analogous in nature for both types of infections; therefore, in the next five figures we only present the results for the super-infection scenario where therapy was applied for 168 days after the equilibrium of infection was obtained. The results here presented are also based on 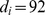 hours, 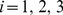, 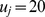 days, 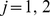, 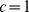, with all other constants specified in the different graphs below. We decided to omit a full discussion based on the effects of the different values considered for the parameters in the antiviral therapy model, due to the fact that the effects of these variations were the same as in the previous section, and therefore we concentrate our discussion on the new parameters of the model, 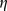 and 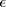.

In the graphs that follow, the vertical dashed lines in the middle graph represent the beginning and the end of the antiviral therapy.

From the graphs, we observe the biphasic linear behavior of HBV viral load as previously reported in [34], [35] For the HBV viral load a marked decrease in the first days of therapy is observed, followed by a slower decrease. For HDV viral load, there is very slow decrease in the beginning of the therapy, which is not surprising due to what is believed about the no direct effect of IFN and LMV in HDV patients. The slow decrease is followed by a marked decrease, which becomes more and more parallel to the viral load of HBV as the efficacy of IFN increases.

**Figure 11:**
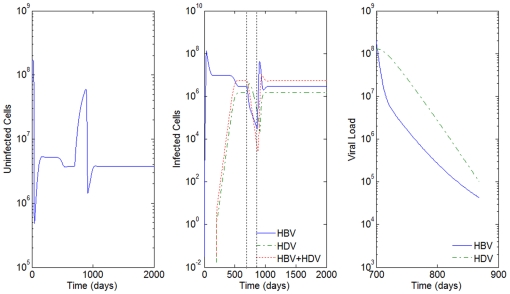
HBV and HDV viral dynamics during antiviral therapy. Number of uninfected cells (left), infected cells (middle), and viral loads during the 168 days of antiviral therapy with LMV alone (right). Initial viral loads of 400 copies/mL, no inhibition of the HBV viral production in cells infected with HBV and HDV and 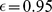.

**Figure 12:**
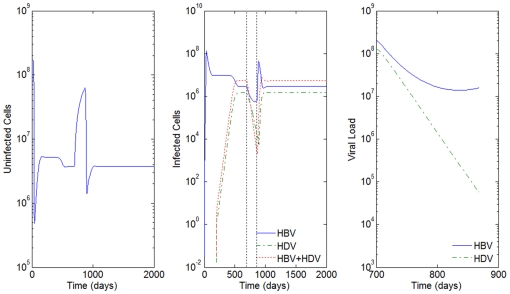
HBV and HDV viral dynamics during antiviral therapy. Number of uninfected cells (left), infected cells (middle), and viral loads during the 168 days of antiviral therapy with IFN alone (right). Initial viral loads of 400 copies/mL, no inhibition of the HBV viral production in cells infected with HBV and HDV and 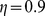.

Figures 11 and 12 represent the dynamic behavior under monotherapy with LMV and IFN, respectively. We observe that even before the end of the therapy period, with only IFN, the HBV viral load starts to increase (right graph of Figure 12). The use of IFN alone seems to have only a momentary effect on the decrease of the infection.

**Figure 13:**
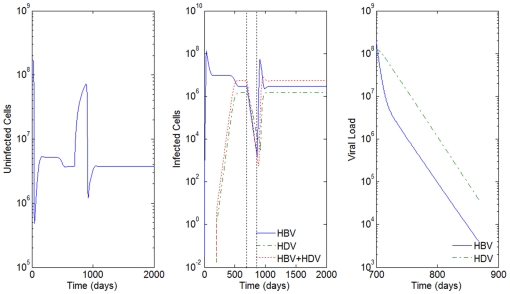
HBV and HDV viral dynamics during antiviral therapy. Number of uninfected cells (left), infected cells (middle), and viral loads during the 168 days of antiviral therapy with LMV and IFN (right). Initial viral loads of 400 copies/mL, no inhibition of the HBV viral production in cells infected with HBV and HDV, 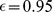 and 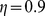.

Notice that when an effective response to IFN therapy is observed (values for 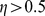), as in Figure 13, the joint antiviral therapy is able to decrease the viral load on an order of 10 at the end of the therapy (from 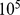 for HDV and 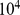 for HBV only with LMV, to 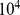 for HDV and 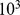 for HBV for the joint therapy).

**Figure 14:**
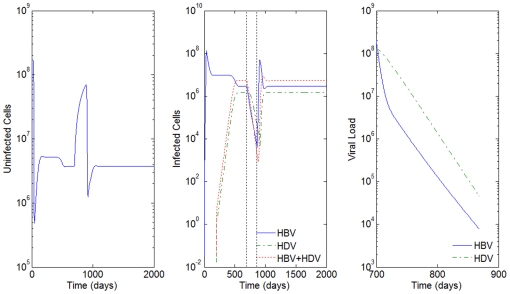
HBV and HDV viral dynamics during antiviral therapy. Number of uninfected cells (left), infected cells (middle), and viral loads during the 168 days of antiviral therapy with LMV and IFN (right). Initial viral loads of 400 copies/mL, no inhibition of the HBV viral production in cells infected with HBV and HDV, 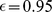 and 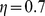.

From the simulation study we realize that as 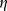 gets closer to 0.5 (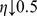) as in Figure 14, the viral load behavior of HBV and HDV infections gets closer to the case when we only apply the lamivudine antiviral therapy (compare Figure 11 to Figures 13 and 14).

**Figure 15:**
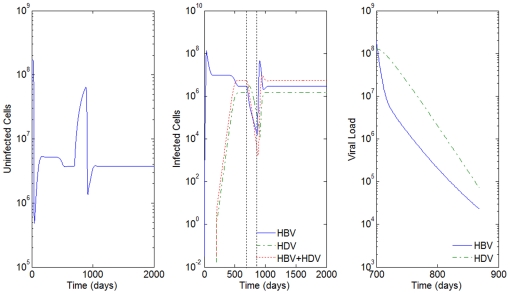
HBV and HDV viral dynamics during antiviral therapy. Number of uninfected cells (left), infected cells (middle), and viral loads during the 168 days of antiviral therapy with LMV and IFN (right). Initial viral loads of 400 copies/mL, no inhibition of the HBV viral production in cells infected with HBV and HDV, 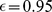 and 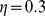.

For the cases where IFN shows little efficacy (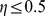), the viral load dynamics of HBV and HDV is similar to the antiviral monotherapy where only LMV is applied, such as in Figure 15.

However, in all cases there is a rebound of the infection when the therapy is terminated. Again, this feature may represent an artifact of this and other similar compartmental ODE models since a complete clearance of the virus is not predicted. However, a rebound of infection after ceasing treatment is a common observation in IFN treated patients (for a review see [36]).

## Discussion

Mathematical models represent useful tools to predict the clinical course of virus diseases and the response to different antivirus therapies. They have been previously used for a number of human viruses, including HBV, HCV, and HIV [19]–[21]. In this study, we describe, for the first time, a mathematical model for HDV infection. Since production of HDV infective particles is dependent on the simultaneous presence of HBV mathematical modeling of HDV infection poses an additional degree of complexity. Both HBV and HDV infect and replicate exclusively in liver cells. Since HDV co-infects or super-infects exclusively HBV infected individuals, the simultaneous behavior of both HDV and HBV and the interaction between the two viruses is considered according to the current knowledge of the biology of the viruses and clinical course of the disease. Little is still known about the mechanisms of HDV replication and its interaction of HBV *in vivo*. The woodchuck model for HDV/HBV infection was able to show some important tendencies on the clinical course of HDV infection, both during co-infection and super-infection. However, in humans the clinical course and response to antiviral therapy may largely differ between individuals and is believed to be also dependent on a number of variables, including the response of the immune system, which is still poorly understood. Since in this work we did not consider modeling the immune response of the host, the obtained results and conclusions shall be interpreted with regard to this limitation.

Six variables were taken into account in the herein proposed model: the number of uninfected cells, the number of HBV infected cells, the number of HDV infected cells, the number of cells simultaneously infected with HBV and HDV, the HBV viral load, and the HDV viral load. Six differential equations were obtained which were subsequently solved using the Matlab software [23], and biological parameters previously described and used by others [24]–[26], [28], [29], [37] when modeling HBV infection. The obtained numerical solutions were consistent with those previously reported by others considering the HBV infection alone [19], [20], [24], [28]. In general, the predicted course of HDV infection is similar to that observed for HBV. Given the same initial viral loads of both viruses we observe a faster increase in the number of HBV infected cells and viral load. After reaching a peak, a small decrease in the HBV viral load and the number of infected cells is observed followed by a stabilization of these parameters of infection with small oscillations around what can be considered as a plateau. Concerning HDV, the increase in the number of infected cells and viral load is slower than the predicted for HBV. Usually, the plateau is reached between 200 and 500 days after infection depending on the initial viral load. In most tested scenarios, the number of HDV infected cells and viral load values remain below corresponding predicted values for HBV. The only exception is observed when an inhibitory factor *c* of HBV replication is introduced during super-infection. This issue is further discussed below. Previous studies aimed to evaluate HBV and HDV activity in infected patients were mainly performed using cross-sectional approaches together with qualitative analysis or low sensitivity quantitative analysis [38]–[40]. This led often to contradictory conclusions with some authors showing that HBV replication may modulate HDV pathogenesis [39], [41], [42] and others claiming that liver disease is mainly due to HDV infection [38], [43]–[46]. To our knowledge, a single quantitative longitudinal study of HBV DNA and HDV RNA dynamics, in 25 chronic patients, has been until now reported [47]. The authors show different replication profiles of HBV and HDV including fluctuating activities of both viruses with alternate predominance across 4–8 year periods of monitorization. Although these oscillations were also predicted by the present model further clinical studies are mandatory to confirm this observation.

Concerning the HDV infection, whether the co-infection or super-infection of HBV infected liver cells, we decided to consider different values for the biological parameters tested. A sensitive analysis was performed taking into account the data reported by other groups concerning both experimental animal infections and monitorization of the course of infection in human patients in the presence or absence of therapy. The parameters tested included the initial viral loads for HBV and HDV, the virion half-life, and the half-life of infected cells. The initial viral loads of an infected individual did not significantly alter the overall course of infection with the number of infected liver cells reaching a plateau which seems to remain stable in the absence of any therapy. The only noticed difference concerns the time needed to reach this plateau which is shorter when the initial viral load is larger. In contrast, the virion half-life and the time infected cells remain alive and thus secreting new infectious virus particles showed to critically influence the course of infection. We found that the combination of these two parameters influences the speed at which the number of HDV infected cells and HDV viral loads reach a plateau. In the case of HBV, it was suggested that the mean life-span of free virions in plasma could vary between 15 and 92 hours and the half-life of infected cells could reach 100 days [28], [37]. Since HBV and HDV share the same envelope proteins we decided to test the same values for the life-span of free virions. The half-life of HDV and HBV infected liver cells was also tested in the range of 10–100 days since we considered that co-infection with both viruses would not increase the half-life of cells when compared with the HBV infection alone. Surprisingly, in our model variations in the values of these two parameters showed to radically influence the possible course of infection. In general, the longer the life-span of free virions the faster the number of infected cells and free virions reaches a plateau. A similar picture was found for the different values of half-life of infected cells tested. Our simulation data suggest that a biological relevant scenario is established for values of half-lives of infected cells above 50 days or 20 days if the mean life-span of free virions will be over 32 hours. Moreover, if the mean half-life of infected cells is below 10 days, then the mean life-span of free-virions should be over 118 hours in order to be possible to observe an increase in the overall number of infected cells and HDV viral load. Since this value is higher than the mean half-life of virions in plasma calculated by others (36.9 hrs; Tsiang and Gibbs [24]) it is possible that a therapy directed to reducing the half-life of infected cells below 10 days would significantly increase virion clearance. Identical pictures were observed for both the co-infection and super-infection scenarios. For values below those indicated by our model it seems probable that a spontaneous clearance of HDV infection would occur. However, to our knowledge, this scenario was not until now observed. It has been previously reported that during the acute phase of super-infection, HDV actively replicates while, at the same time, HBV replication is partially suppressed [48], [49]. The degree of suppression of HBV replication, however, remains largely speculative. We decided to test the influence of HDV suppression of HBV replication by introducing a new variable *c* in our model. The values of *c* tested ranged from 0 (100% inhibition of HBV replication) to 1 (no inhibition of HBV replication). In general, we observed that variations in the values of *c* influenced the predicted relative number of HBV and HDV viral loads and infected cells. For high inhibition values of HBV replication (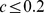) the number of HDV infected cells and HDV viral load surpasses the number of HBV infected cells and HBV viral load, respectively. This picture is not observed when *c* is set to 1 (no inhibition) or when low inhibition of HBV replication is considered (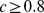).

The potential to predict the behavior of virus infections under the presence of different antiviral therapies is one of the most important issues in mathematical modeling. Accordingly, we decided to test this model giving the most commonly used and generally accepted therapy approaches for HDV infection. These approaches are based on the use of nucleotide analogues, like lamivudine or ribavirine, peggylated 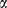-interferon or a combination of both. Nucleotide analogues are known to inhibit HBV replication with efficacies ranging between 90% and 99%, but seem not to have any effect on HDV replication *per se*. The detailed mechanisms of action of interferon-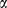 are still controversial but, in any case, it is generally accepted that it induces the expression of a large number of cellular proteins some of which have a direct antiviral effect. Moreover, interferon-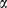 has been implicated in the regulation of adaptative immune responses (reviewed by [50]).

When tested alone, lamivudine showed to be able to reduce significantly the HBV viral load (about 4 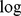 in 150 days) although a complete virus clearance could not be achieved. This may be due to limitations of the model as noticed also by Tsiang and Gibbs [24] when modeling HBV infection alone. The observed reduction was biphasic with a first fast decrease in the beginning of treatment and a second slower in the following weeks. This biphasic behavior was previously reported for LMV treated patients [28]. Although the present model does not predict such a marked biphasic behavior, the decrease in the HBV viral load is clearly faster during the first 15 days of treatment. The biphasic decline of HBV viral load in patients under therapy aimed to inhibit virus production has been previously reported by others (Tsiang *et al.*, [35]). This biphasic behavior was not accommodated by the initial model of HBV viral dynamics developed by Nowak *et al.* [37]. To overcome this problem Tsiang et al (1999, [35]) introduced modifications in the assumptions of the efficacy of inhibition of viral infection. This enabled to show that increasing values of drug efficacy result in an initial faster decline of viral load followed by a slower second phase. The decline during the second phase was found to be similar independent of the values of inhibition tested.

In contrast, concerning HDV, LMV seems to reduce the HDV viral load with less efficiency. In this case, HDV virus clearance is slow in the beginning of treatment and then seems to decay exponentially.

In the case of LMV and interferon-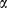 (IFN) combination therapies we tested several scenarios that differed for the efficacy of IFN. For low (30%) and medium (70%) interferon efficacies, the combination therapy did not show a significant improvement in reducing the viral load when compared to the LMV monotherapy. However, if the efficacy of IFN is high (90%) the model predicts a 10 times reduction of HBV and HDV viral loads when compared with LMV monotherapy and low or medium efficacy interferon-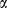 combination therapies. The same pattern was observed for both co-infection and super-infection scenarios. In any case, we could not observe a complete clearance of virus infection after 6 months of therapy. Moreover, after ceasing therapy, a rebound of infection was in all cases observed. Tsiang and Gibbs [24] reported a similar behavior when modeling HBV dynamics alone. This may be due to limitations of the present model since small amounts of free virus particles are predicted to survive in plasma even after prolonged treatment. In any case, we believe that further development of stochastic individual-based models for HDV infection is crucial to clarify this question.

Modeling the scenario for IFN monotherapy showed a significant decrease in the HDV viral load (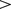
3 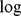 in 150 days) and the number of infected cells. However, in this scenario the HBV viral load did not decrease significantly (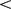
1 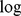 in 150 days) displaying what seems to be a tendency to stabilize at high levels (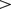 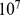 copies/mL).

Finally, both LMV monotherapy and combination therapy of LMV and IFN were predicted to more effectively reduce the HBV and HDV viral loads in the case of super-infection scenarios when compared with the co-infection. In contrast, IFN monotherapy was found to reduce the HDV viral load more efficiently in the case of super-infection while the effect on the HBV viral load was more pronounced during co-infection.

In conclusion, the combination LMV/IFN therapy seems to be more effective in reducing the number of infected cells and viral load of both viruses. LMV alone reduces the HBV viral load faster when compared with HDV, and IFN monotherapy has a significant effect in reducing solely the HDV viral load. In all tested scenarios a rebound of infection could be observed after the end of therapy.

Taken together, this model suggests that there is a need for development of high efficacy therapeutic approaches towards the specific inhibition of HDV replication. These approaches may additionally be directed to the reduction of the half-life of infected cells and life-span of newly produced circulating virions.

Further research is needed to overcome some of the limitations of the mathematical model here proposed; namely, to date, most of the constant parameters in the model are unknown for HDV infections. The present work is just a first step in trying to understand the HDV viral dynamics; however, a more in-depth look is necessary to understand the different behavior regarding co-infection and super-infection. The authors will continue their work of modeling the dynamics of HDV through a hierarchical Bayesian modeling approach. The main difference between this approach and the one here presented, hierarchical Bayesian versus mathematical, is that in a hierarchical Bayesian approach not only are the parameters no longer fixed quantities, but instead random quantities, their distribution depends on additional parameters, called the hyperparameters. The posterior distribution represents the uncertainty of the parameters after taking the data into consideration. MCMC (Markov Chain Monte Carlo) methods allow us to evaluate any characteristic of the posterior by simulating many sample values from it and then approximating any desirable characteristic from its corresponding sample value. The latest developments of free software such as R [51] and WinBUGS [52] overcame some of the difficulties in the implementations of MCMC methods when fitting highly complex models.

Bearing this in mind, and working together with clinicians, we hope in the future to discover additional information regarding viral dynamics of HDV and thus contribute to a better understanding of this pathology during the different treatment therapies.
